# Linear response of mutans streptococci to increasing frequency of xylitol chewing gum use: a randomized controlled trial [ISRCTN43479664]

**DOI:** 10.1186/1472-6831-6-6

**Published:** 2006-03-24

**Authors:** Kiet A Ly, Peter Milgrom, Marilyn C Roberts, David K Yamaguchi, Marilynn Rothen, Greg Mueller

**Affiliations:** 1 Northwest/Alaska Center to Reduce Oral Health Disparities, Department of Dental Public Health Sciences, Box 357475, University of Washington, Seattle, WA 98195, USA; 2 Department of Pathobiology, University of Washington, Seattle, WA 98195, USA; 3 Regional Clinical Dental Research Center, University of Washington, Seattle, WA 98195, USA

## Abstract

**Background:**

Xylitol is a naturally occurring sugar substitute that has been shown to reduce the level of mutans streptococci in plaque and saliva and to reduce tooth decay. It has been suggested that the degree of reduction is dependent on both the amount and the frequency of xylitol consumption. For xylitol to be successfully and cost-effectively used in public health prevention strategies dosing and frequency guidelines should be established. This study determined the reduction in mutans streptococci levels in plaque and unstimulated saliva to increasing frequency of xylitol gum use at a fixed total daily dose of 10.32 g over five weeks.

**Methods:**

Participants (n = 132) were randomized to either active groups (10.32 g xylitol/day) or a placebo control (9.828 g sorbitol and 0.7 g maltitol/day). All groups chewed 12 pieces of gum per day. The control group chewed 4 times/day and active groups chewed xylitol gum at a frequency of 2 times/day, 3 times/day, or 4 times/day. The 12 gum pieces were evenly divided into the frequency assigned to each group. Plaque and unstimulated saliva samples were taken at baseline and five-weeks and were cultured on modified Mitis Salivarius agar for mutans streptococci enumeration.

**Results:**

There were no significant differences in mutans streptococci level among the groups at baseline. At five-weeks, mutans streptococci levels in plaque and unstimulated saliva showed a linear reduction with increasing frequency of xylitol chewing gum use at the constant daily dose. Although the difference observed for the group that chewed xylitol 2 times/day was consistent with the linear model, the difference was not significant.

**Conclusion:**

There was a linear reduction in mutans streptococci levels in plaque and saliva with increasing frequency of xylitol gum use at a constant daily dose. Reduction at a consumption frequency of 2 times per day was small and consistent with the linear-response line but was not statistically significant.

## Background

Xylitol is a naturally occurring sugar alcohol that has been shown to markedly reduce tooth decay. The substitution of xylitol for sucrose in confections and foods may significantly decrease dental caries (for reviews see [1-3]). Published studies and prevention programs using xylitol have followed the dose and frequency of earlier studies which showed reductions in caries and where the daily xylitol dose varied from 4 to 11 g divided into three to five doses and delivered primarily via chewing gum. Studies using dose of 4 to 5 g or less of xylitol per day have reported conflicting results [4,5]. Table 1 summarizes selected published clinical trials where different formulations of xylitol chewing gum were used to evaluate their effectiveness in reducing mutans streptococci and dental caries [see Additional file 1]. These prospective trials were designed to assess the reduction in caries or mutans streptococci levels and not designed to assess the dose or frequency of xylitol use relationship to the reductions.

Isokangas [6] retrospectively divided the subjects of a study into three subgroups by the daily average frequency of self-reported xylitol gum chewing and concluded that caries reduction was observed at consumption frequency of three times per day, but not at 2.5 times or less doses per day. The latter groups did not differ from the untreated controls. Subsequently, Rekola [7] used data from another study to retrospectively assess the frequency-response relationship of xylitol chewing gum and caries. The study concluded that there was a frequency-response relationship where xylitol chewing gum reduces the caries increment (ΔDMFS) with increased frequency of consumption.

This study is the second in a series of prospective studies designed to enhance the science base for the use of xylitol in population-based prevention programs. The initial study showed a linear reduction of mutans streptococci (defined to include both *S. mutans *and *S. sobrinus *species) in plaque and unstimulated saliva to increasing xylitol dose up to 6.88 g/day. The reduction leveled-off between 6.88 g/day and 10.32 g/day. For the smallest dose of 3.44 g/day, a reduction was observed but did not reach statistical significance. These results were consistent at five-weeks and at 6-months. All subjects in the study chewed 3 pieces of xylitol and/or sorbitol gum 4 times per day [8]. This dose-response relationship is in agreement with a recent study conducted in Thailand where a difference between xylitol dose of 5.8 g/day and 11.9 g/day was not observed [9].

The aim of this second study was to prospectively determine the relationship between the frequency of xylitol gum use and mutans streptococci level. This paper investigates the hypothesis that there is a linear reduction in mutans streptococci level in plaque and unstimulated saliva over five-weeks in response to increasing frequency of xylitol chewing gum use at a constant daily dose of 10.32 g.

## Methods

### Subjects

Potential subjects from the Seattle, WA area (n = 352) were interviewed for medical conditions and antibiotic use that would preclude participation. Individuals were excluded if they had taken antibiotics during the last four weeks or anticipated doing so during the study. Subjects with a history of gastrointestinal problems were excluded, as were subjects intolerant to phenylalanine. Screening plaque samples were taken and processed for mutans streptococci enumeration from potential subjects who met the inclusion criteria (n = 337). Of these, 177 subjects with ≥ 10^4 ^CFU mutans streptococci/ml in their sample were invited to participate. An additional six who were screened and met the criteria but were not invited to participate because enrollment had closed by the time their results were available. Among those invited to participate, 75% (132/177) of subjects completed all baseline procedures and were enrolled. The Institutional Review Board of the University of Washington approved this study and the informed consent of the participants was obtained.

### Study design

This prospective randomized, controlled, clinical trial employed a four-group design in which each active group consumed 10.32 g/day of xylitol delivered in chewing gum. The daily 12 xylitol gum pellets were evenly divided into chewing frequency groups of 2, 3, or 4 times/day, denoted as group F2, F3, or F4, respectively. The control group (F0) consumed 12 sorbitol chewing gum pellets evenly divided into 4 times/day. For subjects in the gum use frequency of 4 times/day, study staff and subjects were blinded to their group assignment. Subjects were randomly assigned to groups using a computer generated block randomization procedure. Block randomization was selected to ensure similar proportion of participants in each group. The codes for assignment were kept by the biostatistician and were decoded at the end of the study. Plaque and unstimulated saliva samples were taken at baseline and five-weeks of exposure. Five-weeks was selected because in the first study in this series, mutans streptococci levels were consistent between five-weeks and six months of exposure and because a shorter study period aided recruitment and reduced subject burdens. The study was carried out at the University of Washington Regional Clinical Dental Research Center.

### Gum

Both xylitol and control (sorbitol) gums used in this study were the same as those in the first study in the series [8]. Each xylitol pellet contained 0.858 g xylitol plus gum base, peppermint, menthol, gum Arabic, glycerol, soy lecithin, and glazing agents. Each pellet of the control gum contained the same non-active ingredients plus 0.819 g sorbitol, 0.059 g maltitol, and 0.0015 g Acesulfame K. All gum pellets were formulated by Fennobon Oy (Karkkila, Finland) to be similar in size, consistency, color and sweetness.

### Sample size

The sample size was determined based on intent-to-treat and to provide sufficient power to test the hypothesis that mutans streptococci in plaque and saliva will decrease in response to increasing frequency of xylitol. For the frequency effect analysis, mutans streptococci counts were transformed to log_10 _scale and linear regression was used. A planned sample size of n = 26 subjects per group provided 81% power (2-sided, α = 0.05), where a difference in total mutans streptococci count between the lowest and highest groups was assumed to be log^0.75 ^= 5.6-fold. Assuming a loss to follow-up of 10% to 20%, a total recruitment of 33 subjects per group was necessary to ensure an adequate minimum sample size.

### Adherence

Participants were intensively coached on development of a daily routine for gum chewing and asked to chew the gum for at least five minutes. Pre-packed daily gum packets were distributed at baseline, and after the first and third week of exposure. At each visit, the participant's chewing log was reviewed to monitor compliance and if necessary, an individualized daily gum use schedule was developed and coached based on examination of the participant's daily habits.

Each group chewed 12 pieces of gum per day. To enhance compliance, participants in group F2 (6 pellets each dose, 2 times/day) and group F3 (4 pellets each dose, 3 times/day) were instructed that they could break a dose up into sets of 2 or 3 pellets and chew each set for at least five minutes, one set immediately after the other, and to complete chewing all the gums for each dose within 15 to 20 minutes.

### Plaque and unstimulated saliva sampling

Plaque and unstimulated saliva sampling and laboratory methods were the same as in the first study of the series. First, plaque samples were collected from the cervical third of the buccal surfaces of all teeth using one sterile Kerr applicator per arch and placed in a 5 ml tube containing glass beads and 1 ml of pre-reduced saline. Then, subjects were instructed to let saliva collect without swallowing for at least one minute and then expectorate into the collection tube. This process was repeated as necessary until the minimum 1 ml of saliva was collected. The tubes were immediately carried to the laboratory where they were stored at room temperature until processed generally within three hours. However, samples delivered after 3:30 p.m. were not processed until the following morning (15–17 hours) after delivery. No differences in total counts were observed between samples processed the same day or the following morning. Study staff was trained to conduct plaque and saliva samplings.

### Culture of mutans streptococci

Samples were taken from study participants at baseline and five-weeks after enrollment. Plaque was prepared in pre-reduced saline and 10-fold dilutions of plaque and saliva were prepared separately. For each sample, a modified Mitis-Salivarius agar (Difco Laboratories) supplemented with 500 μg/ml kanamycin, 1% potassium tellurite solution, and 50 U/ml bacitracin (MSKB) was used to enumerate mutans streptococci. The MSKB medium is more specific than MSB for mutans streptococci isolation [10,11] but does not distinguish between *S. mutans *and *S. sobrinus*. Thus, mutans streptococci counts from MSKB plates in this report included both of these species. Only freshly prepared plates were used.

The plaque samples were vortexed to break up the plaque. Then, plaque and saliva samples were diluted. The 10^-0 ^to 10^-3 ^dilutions were plated on MSKB media and incubated in 5% CO_2 _at 36.5°C for seven days prior to enumeration.

In the first study of this series, DNA probes (SSP001 and SM002/SM010) specific for the rRNA variable region of *S. mutans *and *S. sobrinus *[10] were used to verify that only mutans streptococci colonies were counted. Greater than 90% of the randomly selected 1,400 colonies from the MSKB media hybridized indicating that the counted colonies were either *S. mutans *or *S. sobrinus*. This verification process was not repeated in this study.

### Statistical procedures

Linear regression was used to assess the hypothesis of linear frequency-response of mutans streptococci levels after five-weeks of xylitol chewing gum exposure. The linear-response model was based on the formula: Y = ax_1 _+ bx_2 _+ e, where y = log_10 _saliva or plaque at five-weeks, x_1 _= log_10 _saliva or plaque at baseline, x_2 _= frequency (2, 3, or 4 times/day), and e = error. Potential additional terms used in the analyses process includes: x_3 _= dose*dose, to check for nonlinear response, and x_3 _= gender dummy variable or x_3 _= antibiotic dummy variable to determine significance of gender or antibiotics use during the follow-up period. The α-value was set at α < 0.10 as an entry criterion. Where appropriate, descriptive statistics, t-test, analysis of the variance (ANOVA), and least-significant-difference multiple-comparisons test were used to determine differences between the groups at baseline, at five-weeks, and among baseline and five-weeks. The SPSS (v.11.5) and SAS (v.8e) statistical software were used.

## Results

### Baseline

The 132 participants had a mean age of 34 years (range, 18–73), and 57% were women. Participants were 78%, 9%, and 5% Caucasian, Asian, and African American, respectively. Nearly 8% of participants were either Hispanic, Native American or self-identified as mixed race or ethnicity.

The four groups each included 33 participants (n = 132), with females ranging from 42% to 67% in the different groups. Racial distribution among the groups was similar with a predominance of Caucasian (76% to 85%). The mean (range) age of the groups was also similar, 34.5 (19, 70), 34.1 (19, 64), 37.2 (19, 73), 34.1 (18, 60) years for groups F0, F2, F3, and F4, respectively.

There were no statistical differences in mean log_10 _mutans streptococci levels for plaque (F = 0.41, p = 0.75) and unstimulated saliva (F = 0.90, p = 0.45) among the groups at baseline. At the five-week follow-up, 95% (n = 126) of the participants returned for plaque and unstimulated saliva samples collection (F0 = 32, F2 = 31, F3 = 31, F4 = 32). Excluding the dropouts from the analysis did not produce a significant change in the mean log_10 _mutans streptococci counts for each group at baseline.

### Hypothesis testing

**Figure 1:**
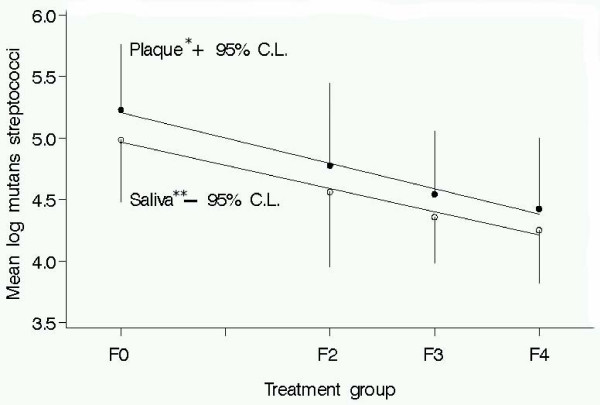
**Mutans streptococci counts in plaque and unstimulated Saliva at five weeks and best fit linear line**. Linear reduction of mutans streptococci levels in plaque and unstimulated saliva to increasing frequency of xylitol chewing gum use at a constant daily dose (10.32 g/day). Linear line equations: plaque – *log mutans streptococci = -.21(Frequency) + 5.21; unstimulated saliva – **log mutans streptococci = -.19(Frequency) + 5.07. Group F0 = Sorbitol Control; F2 = xylitol 2x/d; F3 = xylitol 3x/d; F4 = xylitol 4x/d.

The five-week plaque and saliva samples showed a linear-frequency response where decreases in mutans streptococci levels were associated with higher frequency of xylitol gum chewing at a total daily dose of 10.32 g. The slope of the linear-frequency response was slightly greater for plaque (-0.21) than for unstimulated saliva (-0.19) (Figure 1).

In a sub-analysis, levels of mutans streptococci were significantly different for groups F3 and F4 (p < 0.10) relative to the control group at five-weeks. There was a difference in mutans streptococci levels between group F2 and the control but the difference did not reach statistical significance. Nevertheless, this difference for group F2 was consistent with the hypothesized linear-frequency response model (Figure 1).

## Discussion

This study is part of a series to determine the minimum dose (g/day) and frequency (number of administrations/day) required for xylitol chewing gum to be effective in reducing mutans streptococci levels in plaque and saliva. These studies are important to establish the minimum effective dose and frequency for the use of xylitol in public health programs. Adoption of xylitol use in the United States has been hindered by the absence of such data.

The study design controls for total daily xylitol dose and number of gum pellets chewed. Frequency of xylitol consumption was varied by dividing the total daily numbers of pellets into two, three, or four consumption periods. The results demonstrated a linear-response relationship between the increasing frequency of xylitol gum use and the reduction in the level of mutans streptococci in plaque and unstimulated saliva after five-weeks of exposure. This is consistent with the retrospective studies by Isokangas [6] and Rekola [7] as well as with several other studies where this trend was observed. However, none of these studies was designed to determine the dose- or frequency-response relationships; but rather, they were designed as intervention trials to determine the effect on reduction of caries and/or mutans streptococci levels.

The sub-analysis assessed the differences between test and control groups at each frequency of xylitol gum use. The smaller reduction observed for the group F2 with a frequency of twice per day did not reach significance. However, the observed reduction falls within the range of the hypothesized linear-response slope. Thus, although small, the observed reduction is likely to be real and not by chance. This is also consistent with the retrospective study by Isokangas [6] in which the author concluded that consumption at 2.5 times per day or less produced no significant reduction in caries.

A plateau effect which was observed for xylitol dose reported in the first study of the series was not present in this study. This suggests that although higher frequency maybe more effective in reducing mutans streptococci levels in plaque and saliva, the overall reduction may be limited by the dose plateau. In the Belize intensive intervention trial, the group from the original cohort who had used sucrose gum was selected and given xylitol gum 7 times/day (unsupervised chewing), giving an average daily dose of 14 g. Substantial reductions in both mean DMFS scores and caries rate were observed. However, the author was not able to conclude that the intensive intervention produced better results than those reported in the original study where both dose and frequency of xylitol use were less [12].

This study had several limitations. Plaque collection was not standardized by plaque weight but rather by sampling the arches of teeth. This may be viewed as qualitative and may affect the quantitative analysis. However, the effect is minimized by standardization of sampling and by training of staff. Furthermore, plaque samples at screening compared to baseline and among controls throughout the study did not yield differences in mutans streptococci level. The study used unstimulated saliva to control for mechanical dislodging of mutans streptococci from plaque and falsely or inconsistently increasing mutans streptococci levels.

The two studies in this series thus far confirm what xylitol researchers have speculated for sometime. Studies have shown that xylitol is effective in reducing mutans streptococci in plaque as well as unstimulated saliva (Table 1 [see Additional file 1]). This series of studies as well as other retrospective studies and prospective intervention trials (Table 1) have established that xylitol in the amount of 3.4 g/day or less is unlikely to be effective; amounts greater than 10 g/day are unlikely to yield further reductions; and frequency of exposure of 3 times/day or more is necessary for effectiveness. This knowledge is highly significant in considering the feasibility of public health prevention programs using xylitol.

Xylitol-based caries preventive programs may be more effective in structured daycare centers, Head Start, or at school where the environment offers the best opportunity to achieve compliance. It would be a great challenge to develop home-based prevention programs where a task, any task, consistently requires attention 3 times/day. This is particularly true among the poor and disadvantaged where the stress of day-to-day living already consumes the bulk of their attention. These results also suggest that basic science studies are needed that would help better delineate the mechanisms of action of xylitol so that food products can be engineered to reduce the required frequency and dose of use.

Finally, while xylitol chewing gum at the proper amount and frequency will reduce tooth decay, chewing gum is only a good xylitol vehicle for some populations or age groups. Most school systems in the United States do not advocate for the regular use of chewing gum in the classroom. In many schools, chewing gum is simply prohibited. Autio and Courts [13] found that acceptance of chewing gum in the classroom by teacher was low. In other instances, the children may be too young to safely chew gum. The American Academy of Pediatrics' guidelines for choking prevention listed chewing gum among the foods not to give to children under four years of age. With this in mind, the last two studies in this series will compare the effectiveness of xylitol delivered via different snack foods.

## Conclusion

This study found a linear response relationship between the reduction of mutans streptococci levels in plaque and unstimulated saliva and the increase in frequency of xylitol chewing gum use. The reduction observed for xylitol chewing gum use of 2 times/day was small but consistent with the linear response line. However, this reduction was not statistically different from the sorbitol control.

## Competing interests

The author(s) declare that they have no competing interests.

## Authors' contributions

Dr. Ly was the project director for this series of xylitol studies. Dr. Milgrom was the principle investigator for the studies. Drs. Ly and Milgrom contributed to the design of the studies and were the principle writers of this manuscript. Dr. Roberts was the director of the laboratory that processed the studies' samples and contributed to the studies' laboratory design and procedures and in editing the manuscript. Mr. Yamaguchi was the data manager and analyst. Ms. Rothen supervised and carried out the study procedures and contributed in the editing of the manuscript. All authors read and approved the final manuscript.

## Pre-publication history

The pre-publication history for this paper can be accessed here:



## Supplementary Material

Additional File 1 **Table 1. Brief summary of trials that included xylitol chewing gum and reported effectiveness of xylitol use**. Brief summary of selected randomized clinical trials where xylitol chewing gum was included in the study design and reported effectiveness of xylitol use. The table shows the number of groups in the study, the subjects' age, the frequency and dose of xylitol use and their rationale, the outcome measures, and the conclusions. Click here for file
