# Supplementary material for: Linear response of mutans streptococci to increasing frequency of xylitol chewing gum use: a randomized controlled trial [ISRCTN43479664]
Source: BMC Oral Health. 2006 Mar 24;6:6. doi: 10.1186/1472-6831-6-6 (PMC1482697; doi:10.1186/1472-6831-6-6)
Supplement: Additional File 1 — Table 1. Brief summary of trials that included xylitol chewing gum and reported effectiveness of xylitol use. Brief summary of selected randomized clinical trials where xylitol chewing gum was included in the study design and reported effectiveness of xylitol use. The table shows the number of groups in the study, the subjects' age, the frequency and dose of xylitol use and their rationale, the outcome measures, and the conclusions. [file 1472-6831-6-6-S1.pdf]

## Table 1 – Brief summary of trials which included xylitol chewing gum and reported effectiveness of xylitol use[§]

| Study | Population | Dosing Rationale | Xylitol Frequency | Xylitol Dose (grams/day) | Outcome Measures | Conclusions |
|---|---|---|---|---|---|---|
| 24 months RCT<br>3 groups: Xylitol, Fructose, Sucrose | adult N=125<br>mean Age 27 years<br>dental students | NSS* | mean 4.5 pieces/d (range 3-7) | 6-7 | DMFS | no new DMFS in xylitol group compared to 7 new DMFS in sucrose group [14] |
| 12 months RCT<br>2 groups: Xylitol & Sucrose | adult N=102<br>mean Age 22 years<br>dental students | NSS | mean 4.5 pieces/d (mange 3-7) | 6-7 | DMFS | DMFS decreased by 1 in xylitol group, 3 new DMFS in sucrose group [15] |
| 4 weeks cross-over RCT<br>3 groups: Xylitol, Fructose, Sorbitol/Mannitol | children N=80<br>pedodontic clinic | NSS | 2 pieces, 5x/d non-supervised chewing | 5-7 | saliva and plaque *S. mutans* level | reduction in unstimulated saliva and plaque *S. mutans* level [16] |
| 3 years RCT<br>3 groups: xylitol + xylitol/ fluoride dentifrice, fluoride dentifrice only, no fluoride + restorative only | children N= institutionalized children | NSS | gum and snack foods | 14-20 | DMFS | DMFS increment in the xylitol group = 4.2<br>fluoride group = 6.5, and control group = 7.7 [17] |
| 24 months RCT<br>3 groups: Xylitol 15%, Xylitol 65%, no gum control | children N=433<br>age 8-9 years<br>13 schools | NSS | 1 piece, 3x/d, school days, supervised chewing | xylitol 15% = 0.8<br>xylitol 65% = 3.4 | DMFS increment | DMFS increment<br>xylitol groups = 2.24<br>no gum control = 6.06 [18] |
| Retrospective frequency study<br>Stratified into 3 groups | children N=212<br>age 11-12 years | | 1 piece, 3x/d, non-supervised chewing | 10.5 | change in DMFS | DMFS index decreased with frequency of > 3x/d group. 2.5x/d group no difference [6] |
| 32 months RCT<br>2 groups, xylitol and no xylitol snack foods (including gum) | children N=468<br>age 6-12 | NSS | daily mix of different xylitol snack foods | Maximum 20 (combination of snack foods) | DMFS increment | lower DMFS increment vs. controls. [19] |
| 24 months RCT<br>2 groups: Xylitol vs. no gum | children N=212<br>age 11-12 years | NSS | 1 piece, 3x/d, non-supervised chewing | 10.5 | DMFS increment | lower DMFS increment vs. control[20] |
| Retrospective frequency study | young adult N=100<br>mean Age 22 years | | mean 4.5 pieces/d (range 3-7) | 6-7 | DMFS | reduction in DMFS with increased daily consumption frequency [7] |

**Table 1 (continue)**

| Study | Population | Dosing Rationale | Xylitol Frequency | Xylitol Dose (grams/day) | Outcome Measures | Conclusions |
|---|---|---|---|---|---|---|
| 3 months Field study<br>xylitol gum and tablets freely available without restriction | confectionary workers N=239<br>age 43 year ±10 | NSS | average 7 pieces/d xylitol gum and tablets | Average 3.9<br>(0.45 g/gum piece) | *S. mutans* in stimulated saliva | reduction in number of participants with S. mutans count >$10^6$ CFU/ml [21] |
| 25 days cross-over trial<br>4 groups 3 Xylitol groups,<br>1 Sorbitol control | adult N=20<br>mean Age 26 years (19-37) | NSS | 12x/d | Xylitol 70% = 13.4<br>Xylitol 35% = 6.7<br>Xylitol 17.5% = 3.36 | stimulated and unstimulated saliva and plaque mutans streptococci | higher xylitol level– lower MS counts in plaque and saliva. Xylitol 17.5% no difference vs. control [22] |
| 24 months RCT<br>6 groups: 3 Xylitol, 2 Sorbitol,<br>1 no gum control | children N=510<br>age 6 years<br>10 Schools | NSS | 1 stick or 2 pellets, 5x/d. School or parent supervised | 3:2 xyl/sorb** = 7.1<br>4:1 xyl/sorb = 9.7<br>Xylitol-Stick = 10.4<br>Xylitol-Pellet = 10.7 | caries rate | caries rate reduced in groups chewing gums. 100% Xylitol-Pellet most effective [23] |
| 40 months RCT<br>9 groups: 6 Xylitol, 1 Sorbitol,<br>1 Sucrose, 1 no gum control | children N=1,227<br>age 10 years | NSS | Sticks or pellets 3-5x/d, school days and non-school days chewing | 3:2 xyl/sorb = 5.9<br>1:3 xyl/sorb = 2.0<br>Stick-3x/d = 5.4<br>Stick-5x/d = 9.0<br>Pellet-3x/d = 4.3<br>Pellet-5x/d = 8.5 | caries increment | Sucrose group higher caries than no gum. Fewer caries in xylitol and/or sorbitol gum groups compared to no gum: 100% Xylitol-Pellet most effective [24] |
| 16 months Intensive treatment:<br>1 group: high risk participants | children N=109<br>mean age 13.5 years | Intensive xylitol | 7x/d, mostly non-supervised | 14 | caries onset rate, and DMFS | reduction in caries onset rate, reduction in DMFS scores[12] |
| 3 months RCT<br>3 groups: Xylitol, Sorbitol,<br>No gum control | adults N=164<br>age 36 years ±15 | NSS | 2 pellets, 3x/d non-supervised chewing | not specified (commercially available gum) | stimulated saliva *S. mutans* count | salivary *S. mutans* level in xylitol group lower than sorbitol or control [25] |
| 3 months RCT<br>3 groups: Xylitol 55%, Xylitol 100%, No gum controls. | children N=91<br>age 10-12 years<br>1 school | NSS | 2 pieces, 3x/d School days, supervised chewing | 55% = 5.8<br>100% = 11.9 | stimulated saliva and plaque mutans streptococci | reduction in saliva and plaque mutans streptococci counts [9] |

* NSS = Not specifically stated in the report of the study. Most often, the introduction of the reports may have referenced previous studies with their dosing and frequency. However, the reports did not specifically justify their rationale for choosing their dose or frequency of xylitol use.

** xyl/sorb = xylitol/sorbitol. 3:2 xyl/sorb implies a xylitol/sorbitol ratio of 3:2 in the gum formulation.

§ Machiulskiene et al. [5] reported no significant differences in effect among chewing gum groups in a 5-arms study: [xylitol (0.589g/piece); sorbitol (0.589g/piece); sorbitol/carbamide (0.569g/0.020g/piece); control gum (no polyols); and no-gum group]. Children in gum groups were asked to chew 5 pieces/day, chew for 10 mins/piece preferably after meals. For the xylitol group, 5 pieces = 2.95g xylitol/day. The author concluded that caries preventive effect of chewing sugar-free gum is related to the chewing process. In an Article Analysis & Evaluation, Hayes [26] argued that Machiulskiene et al.'s conclusion was invalid because the study was not an equivalency study designed. Furthermore, xylitol gum was the only group that showed a significant difference compared to no gum even at the low dose of about 3g xylitol/d.
